# Interaction of Protein-like Nanocolloids with pH-Sensitive Polyelectrolyte Brushes

**DOI:** 10.3390/ijms26167867

**Published:** 2025-08-14

**Authors:** Tatiana O. Popova, Ekaterina B. Zhulina, Oleg V. Borisov

**Affiliations:** 1 Center for Chemical Engineering, ITMO University, 197101 St. Petersburg, Russia; salamatovat170301@gmail.com; 2 NRC Kurchatov Institute—PNPI—IMC, 199004 St. Petersburg, Russia; 3 Institut des Sciences Analytiques et de Physico-Chimie pour l’Environnement et les Matériaux, CNRS, Université de Pau et des Pays de l’Adour, UMR 5254, 64053 Pau, France

**Keywords:** nanocolloids, globular proteins, polyampholytes, polyelectrolyte brushes, charge regulation, mean-field theory

## Abstract

The self-consistent field Poisson–Boltzmann framework is applied for analysis of equilibrium partitioning of ampholytic protein-like nanocolloids between buffer solution and weak (pH-sensitive) versus strong polyelectrolyte (polyanionic) brushes with the same net charge per unit area. The position-dependent nanocolloid net charge and the insertion freeenergy profiles are derived as a function of pH and ionic strength in the solution. It is demonstrated that, similar to strong polyelectrolyte brushes, pH-sensitive brushes are capable of the uptake of nanocolloids in the vicinity of the isoelectric point, that is, when the net charge of the colloid in the buffer has either the opposite or the same sign as the ionized monomer units of the brush. At $pI\geq pK_{brush}$ and pH≥pI, the particle absorption patterns by similarly (negatively) charged brushes are qualitatively similar in the cases of strong and weak polyelectrolyte brushes, but the freeenergy barrier at the brush periphery is wider for weak than for strong polyelectrolyte brushes, which may cause stronger kinetic hindrance for the nanocolloid uptake by the brush. A decrease in pH below the IEP leads to a monotonic increase in the depth of the insertion freeenergy minimum inside a strong polyelectrolyte brush, whereas for weak polyelectrolyte brushes, a more peculiar trend is predicted: due to competition between the increasing positive charge of the nanocolloid and the decreasing magnitude of the negative charge of the brush, the absorption is weakened at low pH.

## 1. Introduction

Electrostatically driven interaction and complexation of globular proteins with biological and synthetic polyelectrolytes play a prominent role in nature as well as in many technological and biomedical applications [1]. These assemblies, including immobilization of proteins, enzymes, and antibodies on solid supports, are of particular interest in the fields of biotechnology and medicine. However, when biomolecules adhere to solid surfaces, their biological function might be compromised.

As a promising alternative, immobilization of globular proteins in polyelectrolyte brushes (monolayers of charged macromolecules end-attached to planar substrates or surfaces of colloidal particles, and immersed in aqueous solutions [2,3,4,5,6,7]) has been actively explored over the last few decades [8,9,10,11] (see also ref. [12] for a comprehensive review). It was found that when biomolecules are bound to polyelectrolyte brushes, their enzymatic activity is often preserved [13,14,15,16], which opens a fascinating perspective of using polyelectrolyte brushes in supported enzymatic catalysis.

The most important experimental findings that motivated and guided theoretical research on the interaction of globular proteins with polyelectrolyte (PE) brushes are (i) proteins can be absorbed by both polyanionic and polycationic brushes in the vicinity of the isoelectric point (IEP), i.e., when the charge of the protein globule is not only opposite but also of the same sign as that of the brush-forming chains. Only at $pH_{b}$ sufficiently far away from the IEP proteins are repelled from the similarly charged brush; and (ii) an increase in the ionic strength of the solution suppresses protein uptake by a polyelectrolyte brush on both sides of the protein IEP. These findings unambiguously indicated that protein uptake by PE brushes is electrostatically driven on both sides of the IEP.

While the interaction of proteins with an oppositely charged brush is plausibly governed by Coulomb attraction, the mechanism of protein uptake by a similarly charged brush is more delicate and remains a matter of discussion in the literature. Two possible explanations of this effect have been proposed. According to the first explanation [17,18,19], it is the inhomogeneity of the charge distribution on the protein surface that drives the absorption of proteins by the PE brush. Although the net charge of the protein on the “wrong side” of the IEP is of the same sign as that of the brush-forming chains, patches of charge of opposite sign on the globule surface drive the adsorption of PE chains on these patches, overcompensating for the penalty of polyelectrolyte repulsion from similarly charged patches. Evidently, this mechanism is operative provided that the distribution of anionic and cationic residues on the globule is essentially inhomogeneous.

An alternative mechanism [20,21] is based on the fact that the local pH inside the PE brush differs from that in the buffer solution due to the strong electrostatic field inside the brush. As a result, the net charge of the protein globule (controlled by the balance of ionization of pH-sensitive cationic and anionic residues) may change its sign inside the brush, so that the globule becomes charged oppositely to the brush and is driven inside by the Coulomb force. This second mechanism is more general and does not require any pre-assumption concerning the distribution of cationic and anionic residues on the globule surface. However, as it has been proven by a more accurate theoretical analysis [22,23,24,25], an inversion of the globule charge in the brush is an indispensable but not sufficient condition for the globule uptake by a similarly charged PE brush. As was demonstrated, the charge inversion leads to the appearance of a free energy barrier that may hinder protein uptake by the brush even when it is thermodynamically favorable.

Both experimental and theoretical studies reveal that the buffer $pH_{b}$ is the key control parameter in the interaction of globular proteins and ampholytic protein-like nanocolloids with polyelectrolyte brushes. When the PE chains are negatively charged (polyacid brush), the electrostatic driving force for globule absorption is suppressed under strongly basic conditions ($pH_{b}\gg pI)$. It becomes operative in the vicinity of the IEP, $pH_{b}\geq pI$, and is further promoted upon a decrease in $pH_{b}<pI$. This trend is attributed to the progressively increasing net charge of the nanocolloid — from negative at $pH_{b}\geq pI$ to positive at $pH_{b}<pI$ — in the buffer solution and, with a positive offset, inside the negatively charged brush. The reverse trend is observed for positively charged (polybase) brushes.

In the theory developed earlier [22,23,24,25], it was, however, assumed that the charge on the brush-forming chains does not depend on $pH_{b}$, as it happens for strong (quenched) polyelectrolytes, like polystyrenesulfonate (PSS, anionic) or poly(diallyldimethylammonium chloride) (PDADMAC, cationic). As a result, the electrostatic field created by a quenched PE brush and acting on the ampholytic particle is also independent of $pH_{b}$. In many experiments, however, PE brushes were formed by weak (pH-sensitive) polyelectrolytes, like poly(acrylic) acid (PAA, anionic) [26,27,28] or poly (2-aminoethyl methacrylate hydrochloride) (PAEMH, cationic) [28,29].

Evidently, if $pK_{a}$ of a pH-sensitive polyanionic/polycationic brush is significantly larger/smaller than pI of the nanocolloid, the PE brush is virtually fully ionized in the vicinity of pI. In this case, the interactions of globular proteins with such a brush are similar to those with a strong polyelectrolyte (permanently ionized) brush. The situation may become more delicate if $pK_{a}\approx pI$, that is, variation in pH around the protein IEP is accompanied by a significant change in the ionization of the brush-forming PE chains. One should also bear in mind that due to cooperative intermolecular Coulomb interactions between brush-forming pH-sensitive PE chains, the effective $pK_{a}^{brush}$ is lower (for polyacid) or higher (for polybase) than the “bare” $pK_{a}$ for an individual monomer unit in the buffer and depends on the brush architecture (chain length, grafting density) and the ionic strength of the solution [30,31].

The aim of the present theoretical study is to unravel the effect of variations in buffer $pH_{b}$ on the interactions and uptake of protein-like ampholytic nanocolloids by brushes formed by pH-sensitive (annealing) polyanionic chains with a bare ionization constant $pK_{a}\approx pI$. We aim to investigate how simultaneous variations in the ionization of nanocolloid cationic and anionic residues and of monomer units in the brush-forming chains manifest themselves in the pH-controlled shape of the insertion free energy profiles and thus in the switch from absorption to depletion scenarios. To reach these goals, we implement an analytical self-consistent field Poisson–Boltzmann framework [31,32,33] combined with the numerical Scheutjens–Fleer calculations [34]. The latter approach must be applied under conditions of suppressed ionization of PE chains, i.e., when the free energy contributions due to the electrostatic and the excluded volume intermolecular interactions are comparable.

## 2. Results and Discussion

**Figure 1:**
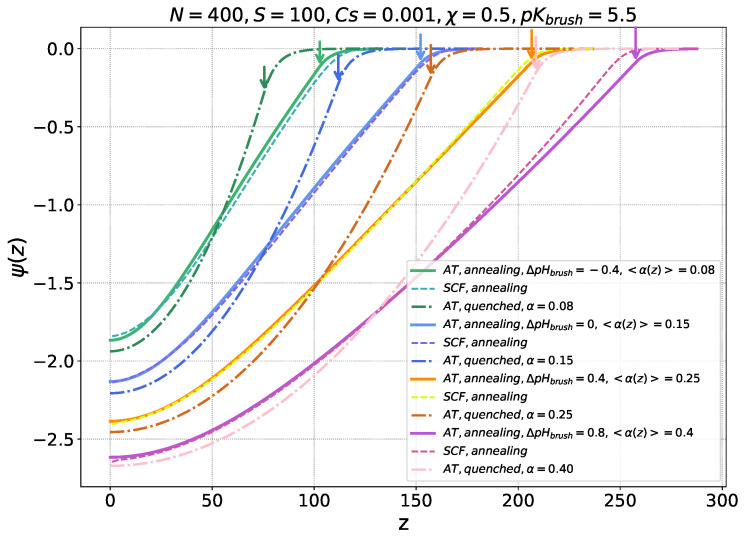
Electrostatic potential profiles for pH-sensitive (annealing) PE brush at different pH offsets $\Delta pH_{brush}=pH_{b}-pK_{brush}$ relative to the brush $pK_{brush}=5.5$. Solid lines correspond to analytical theory predictions, while dashed lines represent numerical results obtained by the Scheutjens–Fleer self-consistent field (SF-SCF) method for the annealing PE brush. Dash-dot lines show electrostatic potential profiles for strong (quenched) PE brushes with average fractions of charged groups α=<α(z)>, matching those of the annealing brush at corresponding $pH_{b}$ values. Other parameters are chain length N=400, grafting area per chain s=100, and salt concentration $c_{s}=0.001$ (in dimensionless units), and solvent quality corresponds to the θ-condition (χ=0.5). Brush boundaries z=H are indicated by arrows in the corresponding colors.

In Figure 1, the electrostatic potential profiles ψ(z) are presented for an annealing (weak) anionic brush at different offsets $\Delta pH_{brush}=pH_{b}-pK_{brush}$ from the brush pK ($pK_{brush}=5.5$) and for a quenched (strong) PE brush with fractions α of permanently charged monomer units equal to the average degrees of ionization <α(z)> of the weak PE brush at each respective value of $pH_{b}$. Four offsets were considered: $\Delta pH_{brush}=-0.4,0,0.4,$ and 0.8 at fixed $pK_{brush}=5.5$. Solid lines correspond to strong-stretching analytical theory predictions, while dashed lines represent numerical results obtained from the Scheutjens–Fleer self-consistent field (SF-SCF) method for the annealing brush. The dash-dot lines represent the electrostatic potential profiles for quenched PE brushes, as obtained from the strong-stretching analytical theory.

As demonstrated by Figure 1, the thickness of the annealing polyanionic brush monotonically increases upon an increase in $pH_{b}$, which is unambiguously attributed to an increase in the average degree of ionization <α(z)> of the monomer units of the brush-forming chains that leads to an increase in the osmotic pressure inside the brush and stronger chain stretching. The local degree of ionization α(z) is an increasing function of the distance *z* from the grafting surface in accordance with Equation (20). For the given choice of the brush parameters and salt concentration, the results demonstrate excellent agreement between the analytical theory (solid lines) and SF-SCF calculations (dashed lines) for the annealing brush with average degrees of ionization <α(z)> between 0.1 and 0.4.

We remind readers that the analytical theory takes into account only electrostatic intermolecular interactions in the brush and assumes linear (Gaussian) elasticity of the brush-forming chains, while numerical SF-SCF modelling accounts for both electrostatic and excluded volume interactions and for the finite chains’ extensibility as well. Therefore, certain discrepancies between the results of the analytical theory and numerical modelling arise: (i) at <α(z)> ≤ 0.1, when the brush is weakly swollen and the contributions of electrostatic and excluded volume intermolecular interactions are comparable; and (ii) at <α(z)> ≥ 0.4, due to the non-linear elastic response of strongly stretched chains (underestimated in the analytical theory). Comparison of the electrostatic potential profiles generated by quenched and annealing PE brushes with the same α = <α(z)> reveals that the quenched brush generates a stronger electrostatic field, which should promote the absorption of ampholytic nanocolloids. Remarkably, as one can see from Figure 1, PE chains in the annealing brush with <α(z)> = α are more extended than those in the quenched PE brush (the annealing PE brush thickness is larger) because the degree of ionization of monomer units increases as a function of *z*, from the grafting surface z=0 towards the edge of the brush z=H.

**Figure 2:**
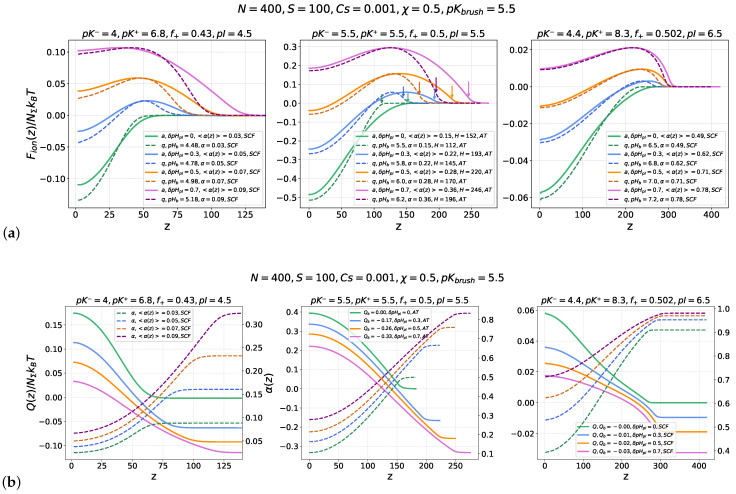
Ionic contribution, $\Delta F_{ion}\left(z\right)/N_{\Sigma }k_{B}T$, to the nanocolloid insertion free energy into a weak (annealing, solid lines) or strong (quenched, dashed lines) PE brush with α=<α(z)> (**a**) and position-dependent net nanocolloid charge $Q\left(z\right)/N_{\Sigma }k_{B}T$ and local ionization degree α(z) of PE chains in the annealing PE brush (**b**) for various values of the $pH_{b}$ offset above the nanocolloid’s isoelectric point, defined as $\delta pH_{pI}=pH_{b}-pI=0,\;0.3,\;0.5,\;0.7$, as indicated in the inserts. The three panels (from left to right) correspond to different values of the nanocolloid isoelectric point pI=4.5,5.5,6.5, corresponding to different fractions ($f_{+}$) and ionization constants of cationic and anionic groups ($pK_{+}$, $pK_{-}$), as indicated above the panels. The ionization constant for the annealing PE brush is $pK_{brush}=5.5$ for all the panels. The position of the brush boundary is indicated by colored arrows in the middle panel. The brush parameters are chain length N=400, grafting density s=100, salt concentration $c_{s}=0.001$, Flory–Huggins parameter χ=0.5 (theta-solvent conditions).

Figure 2 illustrates similarities and differences in the nanocolloid absorption scenario by annealing and quenched PE brushes on the “wrong side” of the IEP, i.e., when the brush and the nanocolloid in the buffer are similarly (negatively, in our case) charged. In Figure 2a, the electrostatic parts $\Delta F_{ion}\left(z\right)/N_{\Sigma }k_{B}T$ of the nanocolloid insertion free energy into an annealing (solid lines) or quenched (dashed lines) PE brushes with a similar average fraction of charged monomer units, α=<α(z)>, are presented. The three panels, from left to right, correspond to different values of the nanocolloid isoelectric point pI=4.5,5.5,6.5, representing nanocolloids with different fractions and ionization constants ($pK_{+}$, $pK_{-}$) of cationic and anionic groups. The ionization constant of the annealing brush, $pK_{brush}=5.5$, is the same in all the panels. Curves of different colors correspond to different offsets $\delta pH_{pI}=pH_{b}-pI$ of the buffer $pH_{b}$ from the respective IEPs of the nanocolloids. For each value of pI, four values of $\delta pH_{pI}$ above the IEP, that is, $\delta pH_{pI}=pH_{b}-pI=0,\;0.3,\;0.5,\;0.7$, corresponding to the zero or negative charge of the nanocolloid in the buffer, are considered.

The profiles of the position-dependent nanocolloid charge $Q\left(z\right)/N_{\Sigma }k_{B}T$ and local brush ionization degree α(z) for the same values of pI and $\delta pH_{pI}=pH_{b}-pI$ are shown in Figure 2b, which illustrates a monotonous increase in Q(z) and a concomitant decrease in α(z) upon approaching the grafting surface.

All three panels in Figure 2a,b demonstrate similar qualitative features in the evolution of $\Delta F_{ion}\left(z\right)$ and Q(z) curves upon an increase in $pH_{b}$ up from the nanocolloid’s IEP for both annealing and quenched PE brushes: At $pH_{b}=pI$, the free energy $\Delta F_{ion}\left(z\right)$ monotonously decreases upon a decrease in *z*, from zero at z=∞ to a negative value attained in the edge minimum located at z=0, which points to thermodynamically favorable absorption of nanocolloids in the brush. Simultaneously, the nanocolloid charge monotonously increases from zero far away from the brush to a positive value inside the brush. The edge minimum of $\Delta F_{ion}\left(z\right)$ at the grafting surface, z=0, is preserved also in the vicinity of the IEP, $\delta pH_{pI}\geq 0$, when the nanocolloid is negatively charged in the buffer, Q(z=∞)≤0, but acquires a positive net charge inside the brush. Additionally, a maximum in $\Delta F_{ion}\left(z\right)$ emerges in the proximity of the brush edge. The position of the maximum coincides with the nanocolloid charge inversion point, pH(z)=pI, as shown in Figure 2b. The height of the maximum increases, whereas the depth of the minimum decreases upon an increase in $\delta pH_{pI}$. Negative values of $\Delta F_{ion}\left(z=0\right)$ indicate the presence of a thermodynamic driving force for the nanocolloid absorption in the brush, whereas positive values of $\Delta F_{ion}\left(z=0\right)$ in the minimum indicate its metastable character, i.e., inversion of the nanocolloid charge upon insertion into the brush does not imply that the colloid absorption by the brush is thermodynamically favorable. These trends in the nanocolloid interaction with a PE brush as a function of $\delta pH_{pI}$ were previously discussed in [23,25].

The depth of the freeenergy $\Delta F_{ion}$ minimum at z=0 is systematically larger for a quenched than for an annealing PE brush for the same α=<α(z)> and the same $\delta pH_{pI}$, which points to more efficient nanocolloid absorption by a strong than by a weak PE brush on the “wrong side” of the IEP. As one can see in Figure 2a, due to the larger thickness of the annealing PE brush than that of the quenched PE brush with the same α = <α(z)>, the freeenergy maximum at the brush periphery is systematically wider in the annealing PE brush than in the quenched PE brush, whereas the magnitude of the maximum for given pI and $pH_{b}$ is the same in quenched and annealing brushes, as follows from Equations (4)–(10).

The right panels in Figure 2a,b correspond to the case $pI=6.5>pK_{brush}$, when the brush is already strongly charged, <α(z)>=0.49 at $pH_{b}=pI$ (i.e. at $\delta pH_{pI}=0$), and its charge further increases upon an increase in $pH_{b}$, reaching <α(z)> = 0.78 at $\delta pH_{pI}=0.7$. As a result of the high ionization degree, the brush-forming PE chains are stretched beyond the linear elasticity regime, and therefore, we use the numerical SF-SCF scheme to calculate the electrostatic potential in the brush. Because in a strongly ionized annealing PE brush at $pH_{b}\geq pK_{brush}$, the local degree of ionization α(z) weakly varies across the brush, the insertion freeenergy $\Delta F_{ion}\left(z\right)$ profiles at $pH_{b}\geq pI$ fairly coincide for quenched and annealing PE brushes with α = <α(z)>. Remarkably, for the chosen values of $pK_{+}$ and $pK_{-}$, the nanocolloid remains very weakly charged in the vicinity of its isoelectric point due to the broad buffering region between $pK_{+}$ and $pK_{-}$, as previously discussed in [25]. As a result, the magnitude of variation of $\Delta F_{ion}\left(z\right)$ is small, and the driving force for the nanocolloid absorption is weak.

The left panels in Figure 2a,b correspond to the opposite case of $pI=4.5<pK_{brush}$, when the annealing PE brush is almost uncharged in the vicinity of the nanocolloid’s IEP (${\langle \alpha \rangle }_{pH_{b}=pI}\approx 0.03$). Therefore, contributions of both ionic and excluded volume interactions between brush-forming chains are essential in determining the grafted chains’ conformations and resulting electrostatic potential profiles, which motivated us to use the numerical SF-SCF approach in this case as well. Since at $pH_{b}\geq pI$, the brush is only weakly charged and the magnitude of the positive charge acquired by the nanocolloid inside the brush is small (see the left panel in Figure 2b), the driving force for the nanocolloid absorption by the brush is weak. As $\delta pH_{pI}$ increases above the IEP, both the colloid and then (at even larger $pH_{b}$) the brush acquire negative charge, leading to mutual repulsion and eventual suppression of the driving force for absorption. Here, the difference between $\Delta F_{ion}\left(z\right)$ curves for the nanocolloid inserted into quenched or annealing PE brushes is most pronounced in the peripheral regions of the brushes, z≤H, with a noticeably wider free energy barrier in the latter case.

The most interesting scenario of the nanocolloid–brush interaction occurs at $pI=pK_{brush}=5.5$, as illustrated by the middle panels in Figure 2a,b. In this case, both the nanocolloid and the brush rapidly change their charge in response to small $\delta pH_{pI}$ shifts with respect to $pI=pK_{brush}$, resulting in the largest magnitude of variation in the electrostatic free energy $\Delta F_{ion}\left(z\right)$ and the most pronounced deviations between $\Delta F_{ion}\left(z\right)$ curves for quenched and annealing PE brushes with the same α = <α(z)>. In particular, this difference is manifested in a significantly wider freeenergy maximum in the case of the annealing PE brush compared to the quenched one. In the $pH_{b}$ range considered here, the chains in the PE brushes are moderately stretched (far below the finite extensibility limit), which enabled us to derive the electrostatic potential and insertion free energy analytically (not shown results of the SF-SCF numerical modelling perfectly match the analytical results).

**Figure 3:**
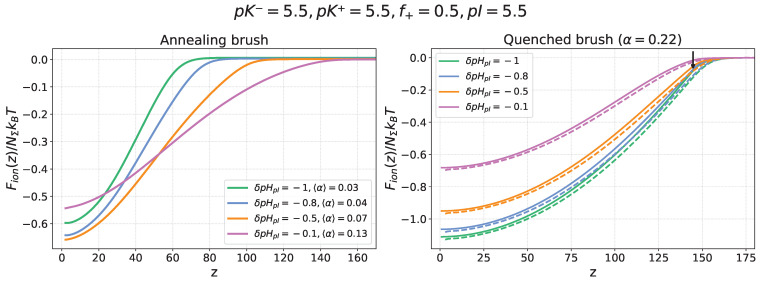
Electrostatic freeenergy $\Delta F_{ion}\left(z\right)/N_{\Sigma }k_{B}T$ profiles of a nanocolloid in a quenched PE brush with α=0.22 (**right** panel) and in an annealing PE brush (**left** panel) at varied offsets $\delta pH_{pI}=-0.1,\;-0.3,\;-0.5,\;-0.8$ (shown by the color code) from the nanocolloid IEP. For the annealing PE brush, variation in $\delta pH_{pI}$ is accompanied by a variation in the average degree of ionization <α(z)>. The ionization constant of the monomer units of the annealing brush-forming chains is set to $pK_{brush}=5.5$, matching the nanocolloid’s isoelectric point (pI=5.5). In the right panel, solid lines represent analytical theory results, whereas dashed lines correspond to the results obtained by SF-SCF numerical modelling. The brush parameters are s=100, N=400, χ=0.5, and salt concentration $c_{s}=0.001$.

Figure 3 presents the electrostatic freeenergy profiles $\Delta F_{ion}\left(z\right)/N_{\Sigma }k_{B}T$ of a nanocolloid in an annealing PE brush (left panel) with a varied, $pH_{b}$-dependent average degree of ionization <α(z)> and a quenched PE brush (right panel) with a fixed degree of ionization α=0.22. The ionization constant of the monomer units in the annealing PE brush is set to $pK_{brush}=5.5$, matching the nanocolloid’s isoelectric point, pI=5.5. The freeenergy profiles are shown for several values of the $pH_{b}$ offset $\delta pH_{pI}=-0.1,\;-0.3,\;-0.5,\;-0.8$, corresponding to acidic conditions ($pH_{b}<pI=pK_{brush}$) when the nanocolloid is positively charged, while the brush is always charged negatively.

Dashed lines in the right panel for the quenched PE brush represent SF-SCF numerical modelling results, which show good agreement with the results of analytical theory (solid lines). For the annealing PE brush (left panel), the freeenergy profiles were obtained by using the electrostatic potential ψ(z), calculated by the SF-SCF numerical method, because the low degree of brush ionization under these conditions requires simultaneous accounting of electrostatic and excluded volume interactions between brush-forming chains.

In the case of the quenched brush, a decrease in $pH_{b}$ below the IEP leads to progressive protonation of the nanocolloid and an increase in its net positive charge, which enhances electrostatic attraction to the permanently negatively charged PE brush and results in an increasing depth of the freeenergy $\Delta F_{ion}\left(z\right)$ minimum inside the brush.

The effect of $pH_{b}$ on the insertion freeenergy $\Delta F_{ion}\left(z\right)$ profiles in the case of an annealing PE brush is more complex. As $pH_{b}$ decreases, the brush becomes less ionized and, as a result, produces a weaker electrostatic field. In spite of the concomitant increase in the positive charge of the nanocolloid, the balance of these two opposite trends may result in a weakening of the electrostatic driving force for absorption at sufficiently low $pH_{b}$. This trend is illustrated in Figure 3 (left panel), where the depth of the free energy minimum, $|\Delta F_{ion}\left(z=0\right)|$, increases upon a decrease in $pH_{b}$ from $pH_{b}=5.5$ to $pH_{b}=5.1$ but then decreases upon further lowering of $pH_{b}$.

**Figure 4:**
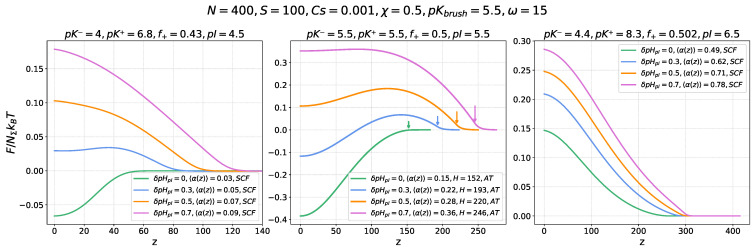
Total free energy $\Delta F\left(z\right)/N_{\Sigma }k_{B}T=\left(\Delta F_{ion}\left(z\right)+\Delta F_{osm}\left(z\right)\right)/N_{\Sigma }k_{B}T$ of the nanocolloid insertion into an annealing PE (polyanionic) brush for various values of the $pH_{b}$ offset above the nanocolloid’s isoelectric point, $\delta pH_{pI}=pH_{b}-pI=0,\;0.3,\;0.5,\;0.7$, as indicated in the inserts. The three panels (from **left** to **right**) correspond to different values of the nanocolloid isoelectric point pI=4.5,5.5,6.5, corresponding to different fractions, $f_{+}$, and ionization constants of cationic and anionic groups ($pK_{+}$, $pK_{-}$), as indicated above the panels. The ionization constant for the annealing PE brush is $pK_{brush}=5.5$. The position of the brush boundary is indicated by colored arrows in the middle panel. The ratio ω of the nanocolloid volume to the number of ionizable groups is set to $\omega =V/a^{3}N_{\Sigma }=15$. The brush parameters are chain length N=400, grafting density s=100, salt concentration $c_{s}=0.001$, and Flory–Huggins parameter χ=0.5 (theta solvent conditions).

Up to now, we have considered only the ionic contribution, $\Delta F_{ion}\left(z\right)$, to the insertion free energy, with a focus on its dependence on $pH_{b}$, and on defining the conditions when the electrostatic driving force for the nanocolloid absorption by the brush emerges. However, even if $\Delta F_{ion}\left(z\right)$ provides the driving force for the absorption, it can be counterbalanced by the osmotic contribution to the free energy, defined by Equation (11). The osmotic term in the free energy arises due to the difference in osmotic pressure of mobile ions in the brush interior and in the bulk of the solution, complemented by excluded volume repulsions between monomer units of the brush-forming chains. The osmotic term is always positive and produces the force expelling the nanocolloid from the brush. For a quenched PE brush, this term is $pH_{b}$-independent, whereas for an annealing PE brush, both the concentration of mobile ions and that of monomer units in the brush depend on its degree of ionization and thus on $pH_{b}$. Figure 4 shows the profiles of the total insertion free energy $\Delta F\left(z\right)=\Delta F_{ion}\left(z\right)+\Delta F_{osm}\left(z\right)$ of a nanocolloid into the annealing PE brush, which includes both electrostatic and osmotic contributions, at three different values of pI and different offsets $\delta pH_{pI}=pH_{b}-pI$ of the buffer $pH_{b}$ above the IEP of the nanocolloid. The sets of parameters used are the same as in Figure 2, not volume. The ratio ω of the nanocolloid volume to the number of ionizable groups is set to $\omega =V/a^{3}N_{\Sigma }=15$.

Comparison of Figure 2 and Figure 4 shows that accounting for the osmotic force expelling the nanocolloid from the brush leads to a systematic shift of the $\Delta F\left(z\right)=\Delta F_{ion}\left(z\right)+\Delta F_{osm}\left(z\right)$ curves with respect to the $\Delta F_{ion}\left(z\right)$ curves upwards, while the shape of the curves in the left and central panels is preserved because of the low or moderate ionization of the brush-forming chains in the vicinity of the nanocolloid IEP and the small excess osmotic pressure in the brush.

For the same values of $\delta pH_{pI}$, the addition of a positive $\Delta F_{osm}\left(z\right)$ term to the $\Delta F_{ion}\left(z\right)$ results in a decrease in the depth of the edge minimum at z=0, or, at larger $\delta pH_{pI}$, to the disappearance of the minimum, while the height of the maximum increases. It is worth mentioning that the position of the total freeenergy maximum does not exactly match the point of the nanocolloid charge inversion.

In contrast, in the right panel, corresponding to $pI>pK_{brush}$, the brush is substantially ionized at $pH_{b}\approx pI$, which produces strong excess osmotic pressure inside the brush. This overcompensates the electrostatic driving force for the nanocolloid absorption even in the proximity of the IEP: the total free energy ΔF(z) monotonously increases upon approaching the grafting surface, and the nanocolloids are expelled from the brush.

**Figure 5:**
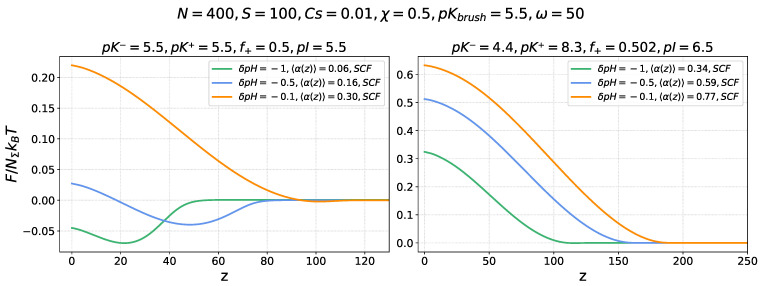
Total free energy $\Delta F\left(z\right)/N_{\Sigma }k_{B}T=\left(\Delta F_{ion}\left(z\right)+\Delta F_{osm}\left(z\right)\right)/N_{\Sigma }k_{B}T$ of the nanocolloid insertion into an annealing PE (polyanionic) brush for various values of the $pH_{b}$ offset below the nanocolloid’s isoelectric point, $\delta pH_{pI}=pH_{b}-pI=-0.1,\;-0.5,\;-1.0$, as indicated in the inserts. The two panels (from **left** to **right**) correspond to different values of the nanocolloid isoelectric point pI=5.5,6.5, corresponding to different fractions ($f_{+}$) and ionization constants of cationic and anionic groups ($pK_{+}$, $pK_{-}$), as indicated above the panels. The ionization constant for the annealing PE brush is $pK_{brush}=5.5$. The ratio ω of the nanocolloid volume to the number of ionizable groups is set to $\omega =V/a^{3}N_{\Sigma }=50$. The brush parameters are: chain length N=400, grafting density s=100, salt concentration $c_{s}=0.01$, and Flory–Huggins parameter χ=0.5 (theta-solvent conditions).

In Figure 5, the profiles of the total insertion free energy $\Delta F\left(z\right)=\Delta F_{ion}\left(z\right)+\Delta F_{osm}\left(z\right)$ are shown for two values of pI=5.5,6.5 and different $pH_{b}$ offsets below the nanocolloid IEPs. In these cases, the nanocolloid is positively charged in the buffer, and the magnitude of its positive charge increases upon insertion into a negatively charged brush, providing an electrostatic driving force for the absorption ($\Delta F_{ion}\left(z\right)$ is negative and monotonously decreases upon approaching the grafting surface). At $pI=6.5>pK_{brush}$ (right panel), the high osmotic pressure in the strongly ionized brush at $pH_{b}\approx pI$ overperforms the electrostatic attraction and suppresses absorption. A decrease in $pH_{b}$ down to $\delta pH_{pI}=-1$ leads to a lowering of the freeenergy profile and the appearance of a very shallow freeenergy minimum close to the brush edge due to the increasing positive charge of the nanocolloid. The same trend is even more pronounced at $pI=pK_{brush}$, when the charge of the brush and, consequently, the osmotic pressure of counterions in the brush rapidly decrease upon lowering $pH_{b}$ below $pI=pK_{brush}$: a shallow minimum in the ΔF(z) curve emerges in the brush periphery even at $\delta pH_{pI}=-0.1$. Upon a further decrease in $pH_{b}$, this minimum becomes deeper and is displaced towards the central region of the brush. Hence, due to the interplay of electrostatic attraction and osmotic repulsion at $pH_{b}$ below the IEP, absorption and accumulation of positively charged nanocolloids occurs in the peripheral and central regions of the negatively charged PE brush, while the nanocolloids are expelled from the proximity of the grafting surface, where the local osmotic pressure is higher.

## 3. Materials and Methods

### 3.1. Nanocolloid Charge and Insertion Free Energy

We consider a protein-like ampholytic nanocolloidal particle of volume *V*, comprising $N_{+}$ cationic and $N_{-}$ anionic ionizable groups on its surface, with respective acidic ionization constants $K_{+}$ and $K_{-}$. The total number of ionizable groups on the particle surface is $N_{\Sigma }=N_{+}+N_{-}$. The fraction of cationic groups is $f_{+}=N_{+}/N_{\Sigma }$. The particle is immersed in an aqueous solution with a fixed $pH_{b}$. (Here and below, the subscript “b” refers to the bulk of the solution.) The solution contains monovalent cations and anions of added monovalent salt, with respective concentrations $c_{+}=c_{-}=c_{s}$, which specify the Debye screening length in the bulk of the solution as $\kappa^{-1}={\left(8\pi l_{B}c_{s}\right)}^{-1/2}$.

The net charge of the particle in the bulk of the solution is given by

(1) $$Q_{b}=\alpha_{b+}N_{+}-\alpha_{b-}N_{-}$$

with

(2) $$\alpha_{b+}={\left(1+10^{pH_{b}-pK_{+}}\right)}^{-1}$$

(3) $$\alpha_{b-}={\left(1+10^{pK_{-}-pH_{b}}\right)}^{-1}$$

being the respective ionization degrees of the particle’s cationic and anionic residues in the bulk of the solution.

The PE chains tethered to the planar surface give rise to an electrical field with an electrostatic potential Ψ(z), depending on the distance *z* from the grafting surface, and calibrated as Ψ(z=∞)=0. The local concentration $\left[H^{+}\left(z\right)\right]$ of hydrogen ions in the brush follows the Boltzmann law,

(4) $$H^{+}\left(z\right)]=\left[H_{b}^{+}\right]\exp\left(-\psi \left(z\right)\right)$$

with $\psi \left(z\right)\equiv e\Psi \left(z\right)/k_{B}T$, which leads to the position-dependent

(5) $$pH\left(z\right)=pH_{b}+\psi \left(z\right)/\ln10$$

and the position-dependent nanocolloid charge

(6) $$Q\left(z\right)=\alpha_{+}\left(z\right)N_{+}-\alpha_{-}\left(z\right)N_{-}$$

with

(7) $$\alpha_{+}\left(z\right)={\left(1+10^{pH\left(z\right)-pK_{+}}\right)}^{-1}$$

(8) $$\alpha_{-}\left(z\right)={\left(1+10^{pK_{-}-pH\left(z\right)}\right)}^{-1}$$

The position-dependent free energy of the nanocolloid is given by

(9) $$\Delta F\left(z\right)=\Delta F_{ion}\left(z\right)+\Delta F_{osm}\left(z\right)$$

The first term in Equation (9) accounts for the ionization free energy of cationic and anionic groups in an external electrical field ψ(z), which can be presented [22,25] as

(10) $$\Delta F_{ion}\left(z\right)/k_{B}T=N_{+}\ln\left(\frac{1-\alpha_{+}\left(z\right)}{1-\alpha_{b+}}\right)+N_{-}\ln\left(\frac{1-\alpha_{-}\left(z\right)}{1-\alpha_{b-}}\right)$$

with the reference state $\Delta F_{ion}\left(z=\infty \right)=0$. The free energy given by Equation (10) can be either positive or negative, depending on the degrees of ionization, $\alpha_{-}\left(z\right),\alpha_{+}\left(z\right)$ of anionic and cationic groups of the colloid, as compared to their ionization degrees, $\alpha_{b+}$ and $\alpha_{b-}$, in the bulk of the solution.

The second term in Equation (9) accounts for the work performed against the excess osmotic pressure upon the nanocolloid insertion in the brush.

(11) $$\Delta F_{osm}=\Delta \Pi \left(z\right)\cdot V$$

The differential osmotic pressure

(12) $$\Delta \Pi \left(z\right)=\Delta \Pi_{ion}\left(z\right)+\Delta \Pi_{pol}\left(z\right)$$

comprises the ideal gas contribution due to mobile ions,

(13) $$\Delta \Pi_{ion}\left(z\right)/k_{B}T=4c_{s}\sinh^{2}\left(\frac{\psi (z)}{2}\right)$$

and the excluded volume contribution due to polymer chains with volume fraction ϕ(z),

(14) $$\Delta \Pi_{pol}\left(z\right)/k_{B}T=-\ln\left(1-\phi \left(z\right)\right)-\chi \phi^{2}\left(z\right)-\phi \left(z\right)$$

where χ is the Flory–Huggins parameter.

Remarkably, $\Delta \Pi_{ion}\left(z\right)$ and $\Delta F_{ion}\left(z\right)$ are non-vanishing beyond the edge of the brush due to the continuity of the electrostatic potential ψ(z). Typically, the first term in Equation (12) dominates over the second one. However, the second term is important for particles embedded in a weak polyanionic brush with $pH<pK_{brush}$.

Here, we introduce the parameter ω, defined as the average nanocolloid volume normalized by the volume of a polymer segment and the number of ionizable groups,

$$\omega =\frac{V}{N_{\Sigma }a^{3}}$$

The relative magnitude of the osmotic and ionic contributions, $\frac{\Delta F_{osm}}{\Delta F_{ion}}$, depends on the parameter ω, since $\Delta F_{ion}∼N_{\Sigma }$ whereas $\Delta F_{osm}∼V$.

### 3.2. Electrostatic Field in Weak and Strong Polyelectrolyte Brush: Analytical Poisson–Boltzmann Approach

To specify the electrostatic potential ψ(z), we consider a brush of ionizable PE chains with a degree of polymerization N, as shown in Figure 1. The grafting density is $\sigma =a^{2}/s$, where *s* is the area per chain on the grafting surface. The PE chains are assumed to be intrinsically flexible, that is, the monomer unit size *a* coincides with the statistical segment length of the corresponding uncharged polymer. The latter is assumed to be on the order of the Bjerrum length $l_{B}=e^{2}/\left(\epsilon k_{B}T\right)$, which equals approximately 7 nm in water under ambient conditions.

In this section, we compare PE brushes formed by strong (quenched) and weak (annealing or pH-sensitive) anionic polyelectrolytes.

Strong PEs possess a quenched (negative) fractional charge αe per monomer unit (with *e* as the elementary charge). In a weak polyacidic brush, every monomer unit is capable of acquiring an elementary negative charge via deprotonation. The fraction α of ionized (deprotonated) monomer units in a weak polyelectrolyte brush depends on $pH_{b}$ in the bulk of the solution and on the local excess electrostatic potential ψ(z) in the PE brush. The maximal value of $\alpha =\alpha_{b}$ is attained infinitely far away from the brush, z→∞ (where ψ(z)→0), and is given by

(15) $$\alpha_{b}={\left(1+10^{pK_{brush}-pH_{b}}\right)}^{-1}$$

Here, $K_{brush}$ is the acidic ionization constant of monomer units in the brush-forming polyacidic chains. A generalization of the theory for PE brushes of positively charged polyelectrolytes is straightforward. Below, we express all lengths in units of *a*, that is, $l_{B}/a\to l_{B}$, $s/a^{2}\to s$, and $c_{s}$ is the dimensionless volume fraction of salt.

To calculate the electrostatic potential ψ(z) created by a weak or strong polyelectrolyte brush, we employ the strong-stretching self-consistent field framework [35], which assumes the parabolic shape of the monomer self-consistent molecular potential U(z) inside the brush:

(16) $$\frac{U(z)}{k_{B}T}=-const-\frac{3\pi^{2}}{8N^{2}}z^{2},\;z\leq H$$

Here, *z* is the distance from the grafting surface, and *H* is the cut-off of the polymer concentration profile (the brush thickness). The constant term in Equation (16) depends on the details of the interactions in the system. Equation (16) is valid for planar or concave brushes of monodisperse flexible chains stretched within the limits of linear (Gaussian) conformational elasticity.

In strong (quenched) PE brushes (subscript “*strong*”),

(17) $$\frac{U_{strong}\left(z\right)}{k_{B}T}=\alpha \psi_{in}\left(z\right),\;z\leq H$$

leading (together with Equation (16)) to the explicit profile of the electrostatic potential,

(18) $$\psi_{in}\left(z\right)=\frac{z^{2}-H^{2}}{H_{0}^{2}}+\psi_{in}\left(H\right),\;z\leq H$$

Here, *H* is the total brush thickness, $H_{0}$ is the characteristic length given by

(19) $$H_{0}=\sqrt{\frac{8}{3\pi^{2}}}N\alpha^{1/2}a$$

and $\psi_{in}\left(H\right)$ should be found from the condition of continuity of the electrostatic potential at the brush edge, z=H.

In a weak (pH-sensitive) PE brush, the ionization degree α(z) of a monomer unit depends on its position *z* in the brush as

(20) $$\alpha \left(z\right)={\left(1+10^{pK_{brush}-pH\left(z\right)}\right)}^{-1}={\left[1+\frac{1-\alpha_{b}}{\alpha_{b}}\exp\left(-\psi (z\right)\right]}^{-1}$$

with Equation (15) specifying $\alpha_{b}$, and Equation (5) for pH(z).

In this case, the molecular potential $U_{weak}\left(z\right)$ is related to α(z) in Equation (20) as [31]

(21) $$\frac{U_{weak}\left(z\right)}{k_{B}T}=\ln\left(1-\alpha \left(z\right)\right)$$

By combining Equations (16) and (21), one finds an explicit expression for the ionization profile in the weak PE brush as

(22) $$\alpha \left(z\right)=1-\left(1-\alpha_{H}\right)\exp\left[\frac{\alpha_{b}\left(H^{2}-z^{2}\right)}{H_{0b}^{2}}\right]$$

with

(23) $$H_{0b}=\sqrt{\frac{8}{3\pi^{2}}}N\alpha_{b}^{1/2}$$

and $\alpha_{H}\leq \alpha_{b}$ being the degree of ionization at the edge of the brush, i.e., at z=H.

The value of $\alpha_{H}$ depends in a complex way on the following parameters of the system: $\alpha_{b}$ (controlled by pH, see Equation (15)), salt concentration $c_{s}$, chain length N, and grafting density $a^{2}/s$. As follows from Equation (22), the degree of ionization α(z) inside the brush monotonously increases as a function of *z*, i.e., with increasing distance from the grafting surface.

By combining Equations (20) and (22), we obtain the electrostatic potential profile, $\psi_{in}\left(z\right)$, inside a weak polyacid brush as

(24) $$\psi_{in}\left(z\right)=-\frac{\alpha_{b}\left(H^{2}-z^{2}\right)}{H_{0}^{2}}+\ln\left\{\frac{1-\alpha_{b}}{\alpha_{b}\left(1-\alpha_{H}\right)}\left(1-\left(1-\alpha_{H}\right)\exp\left[\frac{\alpha_{b}\left(H^{2}-z^{2}\right)}{H_{0}^{2}}\right]\right)\right\}$$

with

(25) $$\alpha_{H}=\left[1+\frac{1-\alpha_{b}}{\alpha_{b}}\exp(-\psi_{in}\left(H\right)\right){]}^{-1}$$

As follows from Equation (24), the electrostatic potential $\psi_{in}\left(z\right)$ inside a weak polyacidic brush follows a more complex functional dependence on *z* than in a strong (quenched) polyelectrolyte brush.

The value of $\psi_{in}\left(H\right)$, or, equivalently, in the case of a weak polyelectrolyte brush, α(H), which is related to ψ(H) by Equation (25), has to be specified from the condition of continuity of the electrostatic potential at the brush edge, z=H, i.e., $\psi_{in}\left(H\right)=\psi_{out}\left(H\right)$.

By assimilating the outer edge of the brush to a uniformly charged plane with charge

$$\tilde{Q}=\int_{0}^{H}\rho_{in}\left(z\right)dz$$

per unit area, with

(26) $$\rho \left(z\right)=-\frac{1}{4\pi l_{B}}\frac{d^{2}\psi_{in}\left(z\right)}{dz^{2}}$$

being the net charge density inside the brush, the distribution of the electrostatic potential outside the brush, at z≥H, can be presented as

$$\psi_{out}\left(z\right)=$$

(27) $$-2\ln\left[\frac{\left(\kappa \tilde{\Lambda }+\sqrt{{\left(\kappa \tilde{\Lambda }\right)}^{2}+1}-1\right)+\left(\kappa \tilde{\Lambda }-\sqrt{{\left(\kappa \tilde{\Lambda }\right)}^{2}+1}+1\right)e^{-\kappa (z-H)}}{\left(\kappa \tilde{\Lambda }+\sqrt{{\left(\kappa \tilde{\Lambda }\right)}^{2}+1}-1\right)-\left(\kappa \tilde{\Lambda }-\sqrt{{\left(\kappa \tilde{\Lambda }\right)}^{2}+1}+1\right)e^{-\kappa (z-H)}}\right]$$

and

(28) $$\psi_{out}\left(H\right)=2\ln\left[\frac{\sqrt{{\left(\kappa \tilde{\Lambda }\right)}^{2}+1}-1}{\kappa \tilde{\Lambda }}\right]$$

Here,

(29) $$\tilde{\Lambda }=\frac{1}{2\pi l_{B}\left|\tilde{Q}\right|}=\left\{\begin{array}{cc} H_{0}^{2}/H^{2} & \alpha =const\\ \alpha_{H}H_{0b}^{2}/\alpha_{b}H & \alpha =\alpha (z) \end{array}\right.$$

is the Gouy–Chapman length outside strong (the upper line) and weak (the lower line) polyelectrolyte brushes.

By using the condition $\psi_{out}\left(H\right)=\psi_{in}\left(H\right)$ and substituting ψ(H) given by Equation (28) into Equation (25), we obtain a closed equation for $\alpha_{H}$ in a weak PE brush as a function of *H*, κ, $H_{0}$, and $\alpha_{b}$ in the form

(30) $$\alpha_{H}={\left(1+\frac{1-\alpha_{b}}{\alpha_{b}}{\left[\frac{\kappa \tilde{\Lambda }}{\sqrt{{\left(\kappa \tilde{\Lambda }\right)}^{2}+1}-1}\right]}^{2}\right)}^{-1}$$

Because mobile cations and anions inside the brush are distributed according to the Boltzmann law,

$$c_{+,in}\left(z\right)=c_{s}\exp\left(-\psi_{in}\left(z\right)\right)$$

$$c_{-,in}\left(z\right)=c_{s}\exp\left(\psi_{in}\left(z\right)\right)$$

the polymer concentration profile inside the brush can be found as

$$c_{p}\left(z\right)=\alpha^{-1}\left(z\right)\left[-\rho_{in}\left(z\right)-c_{-,in}\left(z\right)+c_{+,in}\left(z\right)\right]=\alpha^{-1}\left(z\right)\left[-\rho_{in}\left(z\right)-2c_{s}\sinh\left(\psi_{in}\left(z\right)\right)\right]$$

with the net charge density ρ(z) inside the brush specified by Equation (26), and in the case of a strong PE brush, α(z)≡α.

The result is

(31) $$c_{p}\left(z\right)=\frac{1}{2\pi \alpha l_{B}H_{0}^{2}}[1-\kappa^{2}H_{0}^{2}\sinh\left(\frac{z^{2}-H^{2}}{H_{0}^{2}}+2\ln\left[\frac{\sqrt{{\left(\kappa H_{0}^{2}/H\right)}^{2}+1}-1}{\kappa H_{0}^{2}/H}\right)\right]$$

for a strong PE brush, and

(32) $$c_{p}\left(z\right)=\frac{\alpha_{b}}{2\pi l_{B}H_{0}^{2}}[\frac{1-\left(1-\alpha_{H}\right)\left(1+2\alpha_{b}z^{2}/H_{0}^{2}\right)\exp\left[\alpha_{b}\left(H^{2}-z^{2}\right)/H_{0}^{2}\right]}{{\left\{1-\left(1-\alpha_{H}\right)\exp\left[\alpha_{b}\left(H^{2}-z^{2}\right)/H_{0}^{2}\right]\right\}}^{3}}+$$

(33) $$\frac{\kappa^{2}H_{0}^{2}}{4\alpha_{b}}\left\{\frac{\alpha_{b}}{1-\alpha_{b}}\cdot \frac{\left(1-\alpha_{H}\right)\exp\left[\alpha_{b}\left(H^{2}-z^{2}\right)/H_{0}^{2}\right]}{{\left\{1-\left(1-\alpha_{H}\right)\exp\left[\alpha_{b}\left(H^{2}-z^{2}\right)/H_{0}^{2}\right]\right\}}^{2}}-\frac{1-\alpha_{b}}{\alpha_{b}}\cdot \frac{\exp[-\alpha_{b}\left(H^{2}-z^{2}\right)/H_{0}^{2}]}{\left(1-\alpha_{H}\right)}\right\}]$$

for a weak PE brush.

The average degree of ionization in a weak PE brush can be calculated as

(34) $$<\alpha \left(z\right)>=\frac{s}{N}\int_{0}^{H}\alpha \left(z\right)c_{p}\left(z\right)dz$$

Finally, the brush thickness *H* can be found from the normalization condition

(35) $$\int_{0}^{H}c_{p}\left(z\right)dz=\frac{N}{s}$$

## 4. Conclusions

The interaction of ampholytic protein-like nanocolloids with polyelectrolyte brushes formed by either weak (pH-sensitive) or strong PE chains, end-tethered to a planar surface, and the equilibrium partitioning of nanocolloids between the buffer solution and the brush have been studied using an analytical self-consistent field theory that implements the Poisson–Boltzmann and strong stretching approximations. The electrostatic potential, the position-dependent particle charge, and the insertion free energy were calculated as functions of the particle and brush parameters, as well as the environmental conditions (pH and ionic strength in the solution). Under acidic conditions, when the pH-sensitive polyanionic brush is weakly charged, the electrostatic potential and polymer concentration profiles were calculated using the numerical Scheutjens–Fleer scheme.

The developed theory proves that both strong and pH-sensitive PE brushes are capable of nanocolloid uptake on the “wrong side” of its isoelectric point, that is, when the charge on the colloid particle is of the same sign as the ionized monomer units in the brush-forming chains. Such uptake may be thermodynamically favorable due to the re-ionization of pH-sensitive residues that leads to the inversion of the sign of the particle charge.

However, charge inversion requires overcoming a potential barrier upon particle insertion into the brush. This barrier may sufficiently slow down or even block the particle absorption by a similarly charged brush in the vicinity of the IEP. In the case of $pI\geq pK_{brush}$, the absorption patterns at pH≥pI are qualitatively similar for strong and weak PE brushes, but the insertion free energy barrier at the brush periphery is wider for a weak PE brush.

A decrease in the buffer $pH_{b}$ below the particle IEP leads to a monotonic increase in the magnitude of the positive charge on the particle, which enhances absorption by a strong (permanently negatively charged) brush. However, in the case of a pH-sensitive brush, this trend is counterbalanced by the decreasing degree of brush ionization and the concomitant decrease in the magnitude of the electrostatic potential inside the brush, which weakens and eventually suppresses absorption.

## Data Availability

Data are contained within the article.
